# Circumcision in Males with Bleeding Disorders

**DOI:** 10.4084/MJHID.2013.004

**Published:** 2013-01-02

**Authors:** Hassan Mansouritorghabeh, Abdollah Banihashem, Alireza Modaresi, Lida Manavifar

**Affiliations:** 1 Allergy Research Center, Ghaem Hospital, School of Medicine, Mashhad University of Medical Sciences, Mashhad, Iran.; 2 Haematology Oncology Pediatric Department, Dr. Sheikh Pediatric Hospital, School of Medicine, Mashhad University of Medical Sciences, Mashhad, Iran.; 3 Haematology Lab, Paramedical School, Mashhad University of Medical Sciences, Mashhad, Iran.

## Abstract

**Introduction:**

Male circumcision practice is an invasive procedure that is using worldwide. It makes challenges to haemostatic system and its possible haemorrhagic side effects are more serious in bleeding individuals than normal subjects. In most cases, it can be complete controlled using infusion of appropriate amount of coagulation factors before and post circumcision.

**Aim:**

We aim to documentation type of coagulation therapy and post circumcision practice haemorrhagic presentation among 463 bleeder males of both common and rare bleeding disorders in north eastern part of country.

**Methods:**

We retrospectively gathered information using evaluation medical records in 3 major hospitals during last 15 years and list of patients with bleeding disorders that obtained from haemophilia center. Also a call phone established for each bleeder person to complete data and updating of them. The survey took time from Sep 2009 – Mar 2011. The designed question form included data on doing circumcision or not, types of treatment before and post the procedure and occurrence of bleeding episodes after the surgery.

**Results:**

Overall among 424 cases with various common and rare bleeding disorders who had circumcised, 239 cases (56.3%) had passed the procedure with bleeding experience (indication of undiagnosed cases who underwent circumcision or inadequacy of coagulation therapy), while 185 cases (43.7%) had passed it successfully and without noticeable bleeding experience. The types of coagulation therapy in each group have been cited.

**Conclusion:**

The circumcision practice in unequipped medical center for bleeder ones may make challenges for them and medical services. Also it needed supervision of expert haematologist for

## Introduction

Male circumcision practice is an old surgical procedure that is increasing worldwide.^1^ Historically on ancient Egyptian tombs and old cave paintings, there are evidences based on depictions of circumcision.^2^ Most circumcisions are performed during infancy, childhood and lower in adolescence time.^3^ Circumcision is regarded as an essential procedure for young adults to become a member of society.^2^

The prevalence of procedure performance is affected by religious and cultural associations. According to World Health Organization (WHO) report, it is estimated that about 30% of global male are circumcised that 68% of them are Muslim. While its prevalence is worldwide; it is most common in both Islamic countries and Jewish population. Although circumcision is not directly mentioned in holy Quran, it is generally practiced as to be a custom, while it is practiced as a God’s commandment in Judaism and it is done in eight day of life.^4^,^5^

**Figure 1:**
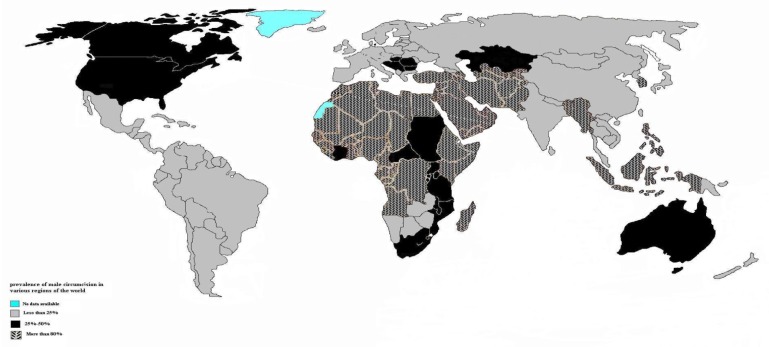
The prevalence of male circumcision practice in various region of the world. Adapted from reference 4.

Also male circumcision is taken up in other parts of world including South and West Africa, New Zealand, United States, and Canada (Figure 1). For example, performance of this procedure has been raised of 48.3% to 61.1% during 1988–1991 in America.^6^

There are some reported benefits regarding male circumcision. They include partial protection against human immunodeficiency virus (HIV),^7^ lower affection by human papilloma virus (HPV), syphilis, cancroid, gonorrhea, possible genital herpes infections^8^,^9^ and lower cervical cancer.^10^ There are also reports that support of this hypothesis in which stated circumcised men encounter lower rate of penile cancer. Also there are data that hold up of prophylactic effect of male circumcision on urinary infection tract.^11^

Circumcision and tooth extraction are two procedures that make commonly challenge to haemostasis system. In the individuals with haemorrhagic diathesis, circumcision practice may encounter patients with life-threatening risk especially in sporadic case without past history of bleeding tendency in the family.^12^ Even when prophylactic regimen of factor VIII is established preoperatively, risk of post-operative bleeding exist.^13^ The post circumcision bleeding is usually treated by supplementary coagulation therapy as systematic approach and although the international guidelines for surgeries in haemophilia has been established, but it is not complete practical in developing countries due to limited availability of factor concentrate.^14^ Also severity of bleeding may vary from dot blot on diaper and oozing until uncontrollable severe bleeding in cases with coagulation defect. In reviewing the literature it seems that post bleeding circumcision in normal subjects have been reported mainly as dot blot and oozing^15^,^16^ while in bleeders it is more complicated.^17^

There are complementary methods such as diathermic knife,^18^ bipolar scissors^19^ and fibrin glue.^20^–^22^ In developing countries where haemostasis abnormalities detection facilities are not enough to cover all part of population, many individuals with bleeding disorders are diagnosed due to excessive bleeding after haemorrhagic episodes. We retrospectively gathered medical data relating to performance of male circumcision practice, bleeding manifestations and types of coagulation therapy in individuals with haemophilia A (HA), haemophilia B (HB), von Willebrand disease (vWD), and rare bleeding disorders in North-eastern Iran.

## Patients and Methods

After approval our study by scientific research committee and giving approval from local ethics committee (by confirmation code: 86679) in Mashhad University of Medical Sciences, we accessed overall 463 male with various inherited coagulation disorders in northeastern Iran. We retrospectively collected data using questionnaire on types of bleeding disorders, age, doing circumcision or not, pre-operative usage of treatment and its type and occurrence of post circumcision bleeding or not. To gather current data, we had a phone call to every affected ones or their parents to fulfill the questionnaire. Also part of medical records in 3 major referral hospitals (Ghaem, Imam Reza and Pediatric Dr. Sheikh) that had been converted to electronic version were evaluated in our province. In current survey post circumcision bleeding regarded positive when the severity of haemorrhage was high and last ≥1 day or needed intervention of medical services, infusion of coagulation or blood products. Oozing and dot blot symptoms were omitted due to many older patients may have forgotten them. The rough data analyzed using SPSS software version 11.5 (SPSS inc., Chicago, IL).

## Results

### Overview

It is estimated that about 1000 individuals with bleeding disorders exist in Khorasan Razavi province.^23^ We could obtain phone numbers of 552 cases in the region. Among them, 462 were males with common and rare bleeding disorders, 423 persons had underwent circumcision (91.5%) and 39 persons (8.5%) did not circumcise (Table 1). They included 284 cases with HA, 89 with HB, 23 with von Willebrand disease, 12 with factor V deficiency, 10 with factor VII deficiency, 2 with factor X deficiency, 19 with combined factor V and VIII deficiency, 16 with platelets disorders, 4 with factor XIII deficiency, 1 with afibrinogemia and 2 with factor XI deficiency.

Overall among 423 cases who circumcised 240 cases (56.7%) had passed the procedure with bleeding experience, while 183 cases (43.3%) had passed it successfully and without noticeable bleeding experience. Post circumcision bleeding has been known as early complication after the circumcision procedure. Its prevalence has been reported as 1–23% in normal infants and children and is depending on used method and experiences of practitioners.^24^

### Haemophilia A

In HA group, 284 cases were identified and entered the study, among them cases 20 (7%) cases had not circumcised. Overall 264 individuals with HA had been circumcised (Table 1). Among them 117 cases (41.2%) had passed circumcision without bleeding. Their mean of age was 25.07 ± 1.44 year old with minimum 3 years, maximum 88 year and mean 23 year. The mean of factor VIII plasma level was 4.19 ± 5.88 % with minimum <1%, maximum 35%, median 1%. They include 89 cases (31.3%) with infusion of factor VIII concentrate, 11 cases (3.9%) using infusion of cryoprecipitate and 17 cases (6%) without usage any coagulation products.

In HA group, 147 cases (51.8%) had passed circumcision with bleeding episodes. Their mean of age was 31.16 ± 1.59 year with minimum 3 and maximum 91 and median 28 year old. Their mean of factor VIII level was 5.26 ± 5.63 % with minimum 1 and maximum 30 and median 3%. They include 132 cases (46.5%) who did not infuse any coagulation products, 12 (4.2%) had infused factor VIII concentrate and 3 (1.1%) had infused cryoprecipitate.

Chi-square test showed there is relation between severity of disease and post circumcision bleeding (p=0.029). The linear association was 0.008 that emphasizes on the lower factor VIII is associated with higher bleeding risk.

### Haemophilia B

In HB group, 89 cases entered the study, 8 cases (9%) had not circumcised and 81 cases had been circumcised (91%). Among them 31 cases (34.8%) had passed circumcision without bleeding (Table 1). Their mean age was 21.90 ± 8.70 year with minimum 6, maximum 45 and median 22 years old. Also their mean of factor IX was 2.61 ± 3.57 % with minimum 1%, maximum 20% and median 1%. There was no relation between severity of disease and post circumcision bleeding, but there was linear by linear association 0.082. They include 29 cases (32.6%) that infused factor IX concentrate pre and post procedure, 1 case (1.1%) had infused FFP and 1 case (1.1%) claimed not to infuse any coagulation products.

On the other hand 50 cases (56.1%) had passed the procedure with bleeding episodes. Their mean of age was 28.26 ± 1.30 year, minimum 5 and maximum 67 and median 27 year old. The mean of factor IX level was 3.97 ± 4.82 %, minimum 1%, maximum 30% and median 2%. They include 43 cases (48.3%) that did not infused any coagulation products, 6 cases (6.7%) that infused factor IX concentrate and 1 case (1.1%) who infused fresh frozen plasma.

### von Willebrand disease (vWD)

Generally 23 male cases with vWD with mean age of 29.04 years old and the youngest was a 11 year old boy and the oldest a 63 year man. They entered the study and all of them had been circumcised (Table 1). There is no data on subtypes of vWD in our patients due to lack of facilities and an expert researcher. Among them 11 cases (47.8%) had experienced bleeding that included 10 cases (43.5%) who did not infused cryoprecipitate or FVIIIC and 1 case (4.3%) who had bleeding despite infusion of cryoprecipitate. Also 12 cases (52.2%) had passed the procedure without bleeding and included 4 cases (17.4%) that infused cryoprecipitate, 4 cases (17.4%) that infused FFP and 4 cases (17.4%) that declared that infused no coagulation products.

### Combined factor V and VIII deficiency (CF5F8D)

19 males with CF5F8D entered the survey. Two of them (10.5%) had not circumcised. Among 17 cases (89.5%) who had been circumcised, 13 cases (68.4%) had bleeding episodes and had not infused any coagulation products and 4 cases (21.1%) had passed circumcision using infusion of FVIIIC and FFP without any obvious haemorrhage (Table 1).

### Platelet disorders (Plt)

16 males with Plt disorders entered the survey, of them 3 persons (18.7%) had not been circumcised (Table 1). We do not have exact subtypes of Plt disorders due to lack of aggregometry and related diagnostic facilities in our region. Among 13 remaining persons (82.4%) who underwent circumcision practice, 7 persons (57.14%) had not bleed and included 6 individuals who had infused Plt bags and a case who claimed had passed the procedure without Plt infusion. Also 6 persons (42.85%) had experienced bleeding that included 5 individuals who had circumcised without infusion of Plt and a case that had bleeding despite infusion of Plt.

### Factor V deficiency

Overall 12 cases with factor V deficiency with mean age of 32 years old came into the study. The younger one was 17 year old and the oldest one was 61 year old. All of them had been circumcised (Table 1). Their mean of factor V level was 6.83% and six of them had severe form. Seven cases (58.3%) had post circumcision haemorrhage that include 6 persons (50%) who had not infuse FFP and a case (8.3%) who had bleeding despite infusion FFP.

Also 5 individuals (41.7%) had passed circumcision without bleeding episodes that included 3 cases (25%) that had not infused FFP and 2 persons (16.7%) that had infused FFP before the procedure.

### Factor VII deficiency (FVIID)

There were 10 males with FVIID with mean age of 18.5 years old that 2 of them (20%) had not circumcised (Table 1). Among 8 circumcised cases the mean of factor FVII was 8.1% and there were 3 cases with severe form (FVII<1%).

Among 8 circumcised cases, five cases had experienced post circumcision bleeding. They included 2 persons (25%) who did not infuse factor VII concentrate or FFP before and after the procedure and had noticeable bleeding. Also 3 individuals (37.5%) who had infused factor VII or FFP concentrate before and after the procedure. One case of current group who had infused factor VII concentrate and experienced bleeding, reported 7 days lasting of haemorrhage after circumcision.

Also among 8 circumcised ones, 3 cases (37.5%) were the patients who had not infused factor VII concentrate and had passed circumcision without noticeable bleeding.

### Factor XIII deficiency (FXIIID)

There were 4 cases with FXIIID with mean of 10.5 years old and 3 of them (75%) had not circumcised. The only individual with FXIIID, who had been circumcised, was a 9 years old child who stated that had passed circumcision with infusion of cryoprecipitate concentrate without bleeding. His factor XIII level was <1% (Table 1).

### Factor X deficiency (FXD)

There were 2 males with FXD with 9 and 56 years old. The younger one with 1% of plasma level of factor X had not circumcised. The older one with factor X level ≈6% had circumcised without infusion of coagulation factor and no bleeding episodes (Table 1).

### Factor XI deficiency

There were 2 brothers with factor XI deficiency without consanguineous marriage in the parents. The younger one was 10 years old with 2% of factor XI level and older was 13 years old with 11% of factor XI. Both of them had passed circumcision practice with infusion of FFP and without obvious bleeding. The discrepancy between plasma levels of factor XI in them is due to inadequate laboratory facilities in our area (Table 1).

### Afibrinogemia

There was a 31 years old case with afibrinogemia (factor XIII < 60 unit) without consanguine marriage in the parents. He underwent circumcision without infusion of any coagulation factor and had bleeding after the procedure (Table 1).

## Discussion

Male circumcision is world widely doing in various countries (both in developing and developed ones). Regarding to its benefits, the procedure operation is increasing noticeably. It seems to be a sort of nearly safe procedure in haemostatically normal neonates and children, due to post circumcision bleeding can be stopped by local pressure. Nevertheless a bleeding’s incidence of side effect between 0.1% and 35% has been reported for healthy boys.^25^ It may be accompanying with serious bleeding complications in neonates and children with unknown bleeding tendency. The haemorrhagic condition may terminate to serious complications, if it will be done in unequipped medical centers. Even in haemostatic health ones it may rarely be associated with infections, incomplete circumcision and needing to suture of dorsal or frenular arteries if it will carry out by traditional circumciser.^26^,^27^ If a child with bleeding tendency undergoes circumcision and encounter bleeding, haemostatic profile study may not detect type of coagulation abnormality, due to the most of coagulation factors decrease below reference values in active bleeding episodes. Acquisition of family history for bleeding tendency before the procedure and doing screening coagulation tests (platelets counts, prothrombin time (PT) and activated partial thrombin time (APTT) tests may be satisfactory indicators of haemostasis system condition. Anyway before circumcision practice, the risks and the benefits of procedure should be discussed with individuals with haemophilia or their parents and it should be stressed that risk of bleeding maybe exists despite replacement therapy before the procedure.

This study showed among 552 known cases, there were 462 male cases that including 83.7% of all bleeding patients had been circumcised that is compatible with report of WHO on circumcision rate in normal population. In our group under study from all bleeding disorders categories, 212 cases had circumcised without infusion of relevant coagulation factors and had encounter post circumcision bleeding. Many of them did not aware of affection to a bleeding tendency in themselves and noticeable numbers of them were sporadic cases without history of affected one in family. This confirms and emphasis on necessity of screening coagulation profile evaluation before circumcision practice.

Also 28 cases (6.9%) with known bleeding disorders (common and rare bleeding disorders) and pre-infusion of coagulation factors or cryoprecipitate or FFP encountered bleeding that may indicate insufficient pre and post treatment with relevant coagulation products infusion, existence of an inhibitory inhibitor or usage of substandard coagulation products. Also it shows the rule of expert haematologist to manage bleeding episodes. While 151 cases had done the procedure with infusion of appropriate amount of coagulation factors concentrate or FFP or cryoprecipitate and did not encounter haemorrhage that emphasis role of consulting with haematologist before doing the circumcision. Thirty two patients claimed that they had been circumcised without bleeding and without usage of coagulation products. They included (17 cases with HA, 1 HB, 4 von Willebrand disease, 3 factor V deficiency, 1 platelet disorders, 3 factor VII deficiency, 1 factor X deficiency and 2 cases with factor XI deficiency). We do not have any clear description for this. There is another possibility that some of the patients have forgotten infusion of coagulation products at time of circumcision practice due to passing many years. In patients with haemophilia who have carrier mother, we know that the factor VIII molecule cannot pass placenta, due to large and huge size of it. The merely description may be that factor VIII is one of C-reactive proteins and it may increase during delivery and other related stress. It has been demonstrated that intensity of bleeding in individuals with factor VII deficiency varies from asymptomatic cases to individuals with severe haemorrhage.^28^

Circumcision is a surgical and invasive procedure that needs to be done in hospital where an experienced surgeon and haematologist exist with sufficient access to adequate dosages of factor concentrate and antifibrinolytic agents and also reliable haemostatic laboratory facilities. This necessity is emphasized for bleeding patients who need to be hospitalized in a medical center with access to haematologist. In current decade the health’s levels has been improved highly in Iran regarding in number of medical centers, scientists and equipments, but it seems more attention needs to be paid to bleeding disorders by health care officials and legislators.

## Figures and Tables

**Table 1:** A review of total number of male individuals with various inherited bleeding disorders and numbers who experienced post circumcision bleeding in each group.

Type of bleeding disorder	Total male Cases	Mean of age ± SD (year)	Total circumcised ones	With post circumcision bleeding			Total in group with post circumcision bleeding	Without post circumcision bleeding			Total in group without post circumcision bleeding
				Without CC infusion	With RCC infusion	With Cryo, Pltb or FFP infusion		With RCC infusion	With FFP, Cryo or Pltb	Without any coagulation factor	
**HA**	284	25.07 **±** 1.44	264	132	12	3	147	89	11	17	117
**HB**	89	21.90 **±** 8.70	81	43	6	1	50	29	1	1	31
**vWD**	23	29.04	23	10	-	1	11	-	8	4	12
**CF5F8D**	19	34.22	17	13	-	-	13	4**	4**	-	4
**Plt**	16	20.27	13	5	-	1	6	-	6	1	7
**FVD**	12	32	12	6	-	1	7	-	2	3	5
**FVIID**	10	18.5	8	2	3	-	5	-	-	3	3
**FXIIID**	4	10.5	1	-	-	-	-	-	1	-	1
**FXD**	2	32.5	1	-	-	-	-	-	-	1	1
**FXID**	2	11.5	2	-	-	-	-	-	-	2	2
**Afibrinogemia**	1	31*	1	1	-	-	-	-	-	-	-
**Total**	**462**		**423**	**212**	**21**	**7**	**240**	**120**	**31**	**32**	**183**

SD; standard deviation, Cryo; cryoprecipitate, RCC; relevant coagulation concentrate, HA; haemophilia A, HB; haemophilia B, vWD; von Willebrand disease, CF5F8D; combined factor V and VIII deficiency, Plt; platelets disorders, FVD; factor V deficiency; FVIID; factor VII deficiency, FXIIID; factor XIII deficiency, FXD; factor X deficiency, FFP; fresh frozen plasma, Pltb; platelet bag,

* age of one patient in the group;

** these are 4 cases with CF5F8D who have infused both FVIIIC and FFP and has been distributed as 2 person in each groups of (with RCC infusion and with FFP or Cryo) to avoid over calculation.
